# Characterization of Hyperreflective Dots by Structural and Angiographic Optical Coherence Tomography in Patients with Diabetic Retinopathy and Healthy Subjects

**DOI:** 10.3390/jcm11226646

**Published:** 2022-11-09

**Authors:** Marie Elise Wistrup Torm, Birgit Sander, Mads Hornum, Paul Krohn, Henrik Birn, Michael Larsen

**Affiliations:** 1 Department of Ophthalmology, Rigshospitalet, Valdemar Hansens Vej 13, 2600 Glostrup, Denmark; 2 Faculty of Health and Medical Sciences, University of Copenhagen, Blegdamsvej 3B, 2200 Copenhagen, Denmark; 3 Department of Nephrology, Rigshospitalet, Inge Lehmanns Vej 7, 2100 Copenhagen, Denmark; 4 Department of Gastroenterology, Rigshospitalet, Inge Lehmanns Vej 7, 2100 Copenhagen, Denmark; 5 Department of Nephrology, Aarhus University Hospital, Palle Juul-Jensens Boulevard 35, 8200 Aarhus, Denmark; 6 Department of Biomedicine, Aarhus University, Wilhelm Meyers Allé 3, 8000 Aarhus, Denmark

**Keywords:** diabetic retinopathy, hyperreflective dots, methodological study, optical coherence tomography, optical coherence tomography angiography

## Abstract

Hyperreflective dots are a common but highly variable feature of optical coherence tomography (OCT) scans of the retina. We studied the spatial characteristics and perfusion of hyperreflective dots using both structural and angiographic OCT B-scans of the macula in 16 eyes in 8 healthy subjects and 8 patients with diabetic retinopathy without macular edema. Hyperreflective dots were manually graded in a 1000 µm parafoveal area by number, diameter, location and perfusion status and traced through adjacent B-scans at 11 µm intervals to determine their length. Thereby, this study defined a procedure to identify granular and elongated hyperreflective elements and differentiate between presumably perfused and occluded capillaries. The latter were only found in the diabetic patients. This classification can potentially be automated to non-invasively identify capillary non-perfusion in vivo.

## 1. Introduction

The retina can be examined non-invasively at a micrometer resolution using clinically applicable imaging methods. Optical coherence tomography (OCT) has the virtue of providing three-dimensional images of tissue reflectivity with information that is largely inaccessible by other methods, such as fine granulation that varies with location and time [1,2,3]. The present study examined a feature of granularity called hyperreflective dots, which occur in both health and disease and which are of a size comparable to that of a retinal capillary in cross-section [4].

Little is known about the ultrastructural and biological characteristics of hyperreflective dots. Previous studies have suggested that the dots share characteristics with small aggregates of hard exudate [1,5], but they have also been found in the complete absence of exudative processes, in conditions where their distribution, density, and relative regularity of size supports that they have a non-exudative origin [6,7]. Moreover, their size and behavior suggest that they may represent cells that are capable of moving through the retina [3,8], such as macrophages, which are inherently mobile [9], and microglia, which may assume amoeboid characteristics [10,11].

The aim of this study was to perform a manual assessment of stacks of closely spaced structural and angiographic OCT B-scans of the retina to analyze the spatial relations and perfusion characteristics of hyperreflective dots in healthy subjects and patients with diabetic retinopathy without macular edema. This assessment showed that hyperreflective dots in the vascular inner nuclear layer primarily represented capillaries, whereas dots in the avascular outer nuclear layer represented granular structures.

## 2. Study Subjects and Methods

We performed a retrospective analysis of OCT scans of diabetic patients referred for retinopathy evaluation between April and July 2018. For comparison, healthy subjects who underwent OCT in relation to routine clinical examination were included. The analysis was cross-sectional, including one visit and one eye per participant. The study was approved by the review committee of the Head and Orthopedics Center of the Rigshospitalet and followed the guidelines of the Helsinki Declaration. All participants gave their written informed consent.

The inclusion criterion for patients was type 1 diabetes and diabetic retinopathy [12], including patients with a history of kidney failure being under evaluation for or having undergone combined pancreas–kidney transplantation. The inclusion criterion for healthy subjects was self-reported good systemic and ocular health without prior eye disease. Healthy subjects were selected to approximately match the age of each patient. The exclusion criteria for the study eye of all study subjects were macular edema, fundus photographically visible hard exudate in the macula, vitreous hemorrhage, fibrotic proliferations in the macula, prior vitrectomy, photocoagulation scars within 2 optic disc diameters of the center of the fovea, and poor quality of fundus images or OCT scans.

Clinical examination of the diabetic and healthy participants included refractioning, an assessment of best-corrected Snellen visual acuity, 50° color and red-free fundus photography (TRC-50DX Mydriatic Retinal Camera, Topcon Corporation, Tokyo, Japan) and OCT (Spectralis OCT-2, 85 kHz, version SP 6.7a, Heidelberg Engineering, Heidelberg, Germany, super-luminescence diode emitting infrared at a center wavelength of 880 nm and a bandwidth of 50 nm, nominal axial resolution 7 µm, lateral resolution 14 µm). Structural OCT was performed as 131 horizontal B-scans spaced 11 µm apart over a 15-degree-by-5-degree (4.5 × 1 mm) fovea-centered field in high-resolution mode with a maximum real-time averaging of 100 frames per B-scan. Angiographic OCT was performed with the best achievable overlapping scan type as 512 horizontal B-scans spaced 5.9 µm apart over a concentric 10-degree-by-10-degree (3 × 3 mm) fovea-centered field in high-resolution mode with a maximum real-time averaging of 7 frames per B-scan. All scans were handled using the manufacturer’s proprietary software (Heidelberg Eye Explorer HEYEX, version 1.10.2.0, Heidelberg Engineering, Heidelberg, Germany) and Microsoft Power Point.

### 2.1. Data Analysis

The analysis comprised the inner and outer nuclear layers of the retina, where hyperreflective dots stand out in contrast to the relatively hyporeflective background. The outer nuclear layer contains no vessels in healthy eyes and rarely in disease either, whereas in the inner nuclear layer capillaries are abundant. Most macular capillaries on either side of the fovea run a vertical course and are therefore mostly seen in cross-section on horizontal B-scans. A dot seen on a single B-scan may represent a granular structure, of which the three-dimensional shape is spherical or quasi-spherical, or an elongated element, notably a capillary, of which the three-dimensional shape appears string-like at the currently available OCT resolution. Angiographic OCT adds motion contrast signals indicating blood flow that can be used to assess if a presumed capillary is perfused or not, of which the latter is common in diabetic retinopathy [13,14].

**Figure 1:**
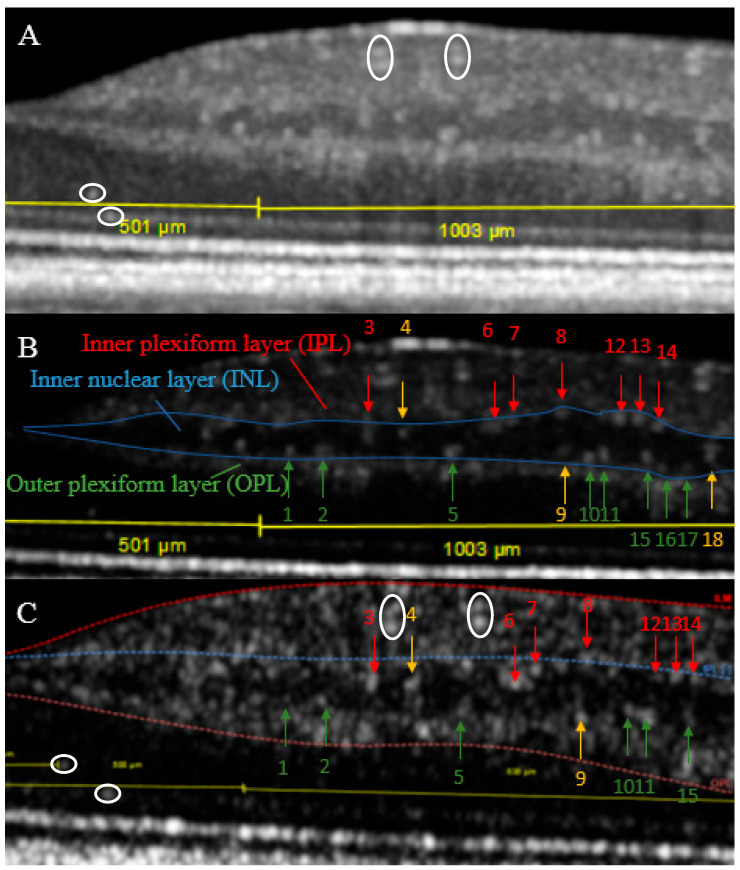
Structural OCT B-scan from an eye with diabetic retinopathy (patient no. 1) in default contrast mode (**A**) and with contrast suppression (**B**). In the latter, colored arrows indicate selected hyperreflective dots in the inner nuclear layer in a zone 500–1500 µm from the center of the fovea (green: location near OPL, red: location near IPL, yellow: location within the INL). Also shown is the same section through the retina as seen on the corresponding structural B-scan of the angiographic OCT, for comparability shown without motion contrast signals (**C**), again with contrast suppression (numbered arrows indicate retrieval of a specific hyperreflective dot on both scans). The white circles in (**A**,**C**) indicate anchoring points for the correlation between the two types of B-scans. The signal-to-noise-ratio is higher within (**B**) (averaging of 100 scans) than in (**C**) (averaging of 7 scans). Figure 5 shows (**C**) with motion contrast signals and the classification of dots.

A full analysis of the inner nuclear layer was performed on the basis of the manual segmentation of a single transfoveal structural OCT B-scan of high quality and its adjacent scans. In the inherent OCT software, the B-scan in question was divided into a region 500 to 1500 µm temporal from the center of the fovea, thus excluding the foveal avascular zone (Figure 1A) [6]. The image contrast was decreased to diminish background speckle noise. Then, the hyperreflective dots within the 1000 µm parafoveal region were marked and counted (Figure 1B). The diameter of a given dot was measured on the B-scan in the axial direction. Any extensions of the dot into the adjacent layers in the transversal dimension (i.e., in the vertical anatomical direction) were followed, and its length could thereby be estimated as 11 µm times the number of adjacent B-scans. Obviously, the real length could be nearly 22 µm longer, but for simplicity we preferred the conservative estimate. The location of a dot was deemed to be near the inner or outer plexiform layers if it was attached to either of these, or inside the inner nuclear layer if its full circumference was free of contact with these adjacent layers (Figure 1B).

For the outer nuclear layer, all 131 consecutive structural OCT B-scans were inspected for hyperreflective dots. The counts included only dots of diameters larger than 15 µm and a distance from adjacent layers that was greater than the width of the individual dot. Thus, the grading excluded localized thickening of the outer plexiform layer or the outer limiting membrane.

For examination of perfusion status of each hyperreflective dot, structural and angiographic OCT B-scans were aligned manually. Instrument constraints made it impossible to acquire structural and angiographic OCT B-scan blocks with identical location and spacing. Therefore, selection of the angiographic B-scan with a location most similar to that of the transfoveal structural B-scan was based on landmarks on the corresponding infrared fundus images followed by identification of shared characteristics in the B-scans, such as location of large and small vessels in cross-section and of hyperreflective dots (Figure 1A,C). Thus, a satisfactory alignment could be achieved within a tolerance of ±1 B-scan of the angiographic B-scan block. The two types of B-scans were scaled and cropped in Microsoft Paint and inserted into Microsoft Power Point. Here, it could be assessed if a given dot in the structural B-scan was overlayed by motion contrast signals or not in the angiographic B-scan. Furthermore, it was evaluated whether an anteriorly located vessel could give rise to a projection artifact. Additionally, in the inherent OCT software, the perfusion of dots spanning adjacent scans was examined for each of these scans using an intensity-bar to adjust the presence of the motion contrast signals overlaying the structural B-scan of the angiographic OCT. This manual data analysis was performed by a single operator (MWT).

### 2.2. Classification

Hyperreflective elements spanning up to, but no more than three adjacent B-scans, corresponding to a minimum transverse extent of 22 µm and a maximum of <44 µm, were classified as granules. Hyperreflective elements spanning more than three adjacent B-scans, hence being approximately 33 µm or longer, were classified as elongated. The justification for setting the limit to three adjacent B-scans was to include all elements with potentially similar axial and transversal dimensions of up to 30 µm in the classification of granules [4]. Furthermore, based on the presence or absence of a motion contrast signal, each hyperreflective element was classified as a granular element; a perfused filiform (string-like) element; or a non-perfused filiform element. If the motion contrast signal was consistently located adjacent to the element, then it was interpreted to be non-perfused. If a dot on the structural B-scan could not be identified on the angiographic B-scan, or if there was an inconsistency in its spatial characteristics between the two types of scans, then it was unclassifiable.

### 2.3. Statistics

The study was performed for the purpose of developing and evaluating a method of manual image analysis that can potentially be used to program an automated image analysis algorithm. For descriptive purposes, the non-parametric Wilcoxon test was used to calculate *p*-values, and no correction was made for multiple sampling. Results are shown as median and/or range.

## 3. Results

### 3.1. Study Subjects

Of the eight patients with type 1 diabetes (age 29–66 years (median 47.5 years) and diabetes duration 17–44 years (median 26.5 years)), five had mild non-proliferative diabetic retinopathy (NPDR) and three had, 8–20 years previously, undergone panretinal photocoagulation sparing the macula for proliferative diabetic retinopathy (PDR) [12] (Table 1). If both eyes were eligible according to the inclusion and exclusion criteria, that from which the image quality was highest was selected. All eight study eyes in the eight patients with diabetes had little or no retinopathy activity (few mild retinal hemorrhages or microaneurysms, but no hard exudates in the macula or neovascularization), and no macular edema had been described previously. Thus, there were no exudative retinal lesions that were likely to be a source of hyperreflective dots. The best-corrected Snellen visual acuity in the study eye was 20/25–20/16 (median 20/20). The study also included one eye in each of eight healthy subjects (age 23–63 years (median 47.5 years)). Healthy subject no. 9 was approximately age-matched to patient no. 1, healthy subject no. 10 to patient no. 2, etc. (Table 1).

### 3.2. Hyperreflective Dots

The number of hyperreflective dots in the inner nuclear layer in the 1000 µm parafoveal area of one B-scan varied from 7 to 18 (median 13) in diabetic patients and from 6 to 16 (median 9.5) in healthy subjects (Table 2). Thus, the density of dots was on average comparable in the two groups. There was no difference in number between mild NPDR and quiescent PDR, and no correlations with age, visual acuity, or diabetes duration.

**Figure 2:**
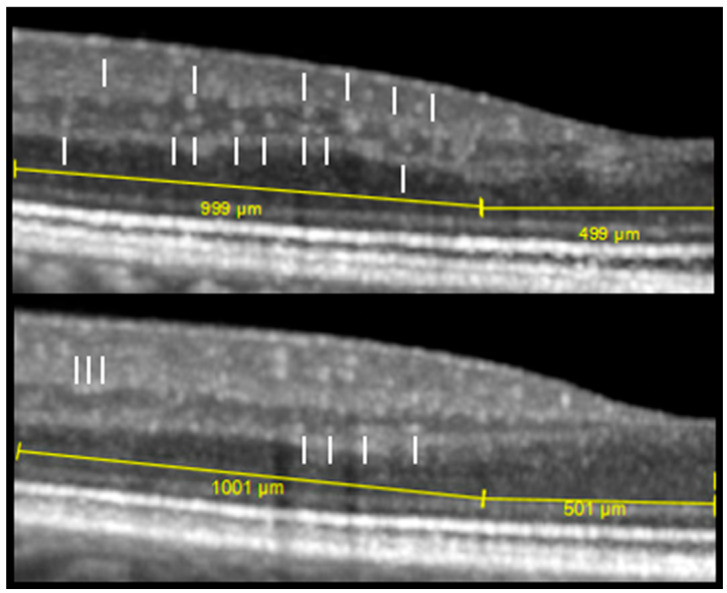
Image example of the 1000 µm parafoveal zone of horizontal transfoveal OCT B-scans from patient no. 8 with quiescent proliferative diabetic retinopathy (PDR) (**top**) and healthy subject no. 16 (**bottom**). The diabetic patient had larger and more irregularly distributed hyperreflective dots (white lines) in the inner nuclear layer of which many were at some distance from the outer and inner plexiform layers compared to the healthy subject.

**Figure 3:**
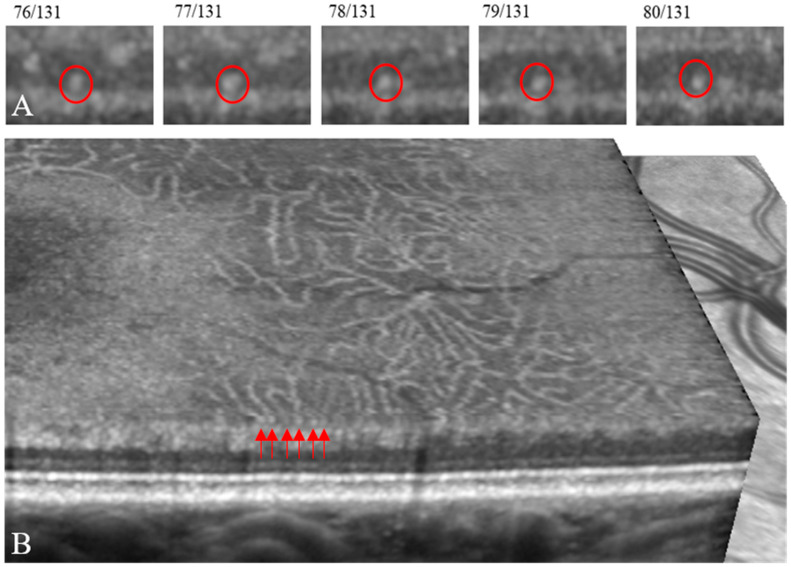
(**A**) Structural OCT B-scans at 11 µm intervals show the trace of a hyperreflective element that was first encountered in B-scan 76 out of 131 (left, red circle), from where the structure could be followed through four more adjacent scans, over a distance of 44 µm, where it seemed to give off a branch to the right (pt. 1, dot no. 5 in Figure 1). The total length of the element was 88 µm and its mean diameter 19 µm, and thus it is a prototype filiform element. By alignment with the angiographic OCT B-scan it was shown to be perfused (see dot no. 5 in Figure 5). (**B**) In the OCT software, the module “3D View” also illustrated that individual hyperreflective dots in a B-scan (above red arrows) in the inner nuclear layer, here located near its outer border, mainly represented capillaries seen in cross-section, here from the deep capillary plexus (seen en face) (example from co. 16).

On single B-scans, the axial diameter of hyperreflective dots was between 10 and 25 µm. A more precise diameter could not be measured reproducibly in the inherent OCT software, but hyperreflective dots in the inner nuclear layer appeared larger in patients with diabetic retinopathy than in healthy subjects (Figure 2). The main part of the dots spanned more than three adjacent B-scans and hence were classified as elongated (on average 10/13 dots in diabetic patients and 8/10 dots in healthy subjects), and their transverse dimension ranged from 51 to 183 μm in diabetic patients and from 58 to 128 μm in healthy subjects (Table 2). Occasionally, an elongated element followed over adjacent B-scans could be seen to branch into two separate dots, as would be expected of a capillary (Figure 3A). Additionally, the three-dimensional view in the software supported that hyperreflective dots in the inner nuclear layer mainly represented cross-sections of the capillary plexuses (Figure 3B). A minor proportion of the dots (on average 3/13 dots in diabetic patients and 3/10 dots in healthy subjects) were smaller than approximately 33 µm in the transverse direction and classified as granules (Table 2).

Hyperreflective dots in the inner nuclear layer were mostly located near the outer plexiform layer in five out of eight diabetic patients and in eight out of eight healthy subjects (Table 2). The second most common location was near the inner plexiform layer. The dots located inside the inner nuclear layer were significantly more common in diabetic patients than in healthy subjects (*p* = 0.013, Table 2), especially in quiescent PDR.

**Figure 4:**
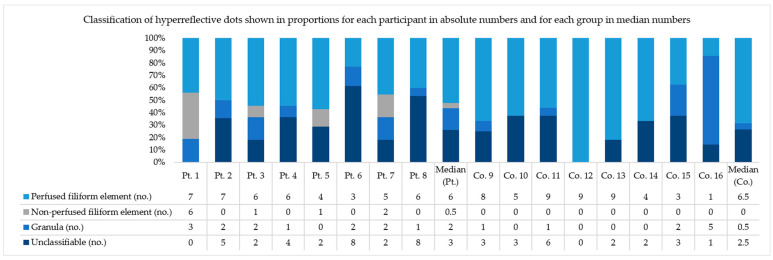
Classification of hyperreflective dots in a 1000 µm parafoveal area of the inner nuclear layer of the retina by analysis of a transfoveal OCT B-scan and its adjacent scans from a single eye per study participant (from a block of 131 B-scans spaced 11 µm apart covering a 4.5 × 1 mm area) and alignment with corresponding angiographic OCT B-scans (from a block of 256 B-scans spaced 5.9 µm apart covering a 3 × 3 mm area). Note, the difference in scan width excludes some of the dots from the structural scans in Table 2. Abbreviations: Pt.: diabetes patient with diabetic retinopathy. Co.: Healthy control subject. No.: number. “Perfused filiform element” indicates the presence of a given hyperreflective dot in more than three adjacent B-scans overlayed by a motion contrast signal, “Non-perfused filiform element” indicates the presence of a given hyperreflective dot in more than three adjacent B-scans without a motion contrast signal, and “Granula” indicates the presence in three or less adjacent B-scans without a motion contrast signal. “Unclassifiable” means that there is inconsistency in the appearance of the dot between the structural and angiographic B-scan. The differences in proportions of the classified dots between diabetic patients and healthy controls were not significant (*p*-values 0.69, 0.11, 0.48, and 0.28, respectively).

**Figure 5:**
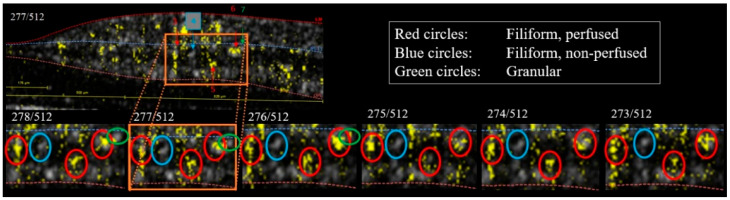
Transfoveal, horizontal, angiographic OCT B-scan showing structural information in grey with overlaid motion contrast signals in yellow from a patient with diabetic retinopathy (**top half**; pt. 1). A selected area (orange box) is magnified (**bottom half**) and shown together with five adjacent B-scans spaced 5.9 µm apart (bottom). Hyperreflective dots seen to extend through the stack of scans with an overlying motion contrast signal (red circles) constitute perfused filiform elements, while such a dot without any motion contrast signal (blue circles) represents a non-perfused filiform element. A hyperreflective dot that can be followed only through two adjacent B-scans and has no motion contrast signal represents a granular hyperreflective element (green circles).

The proportion of hyperreflective elements on the structural OCT B-scans that could be classified by alignment with the corresponding angiographic B-scans was 68.5% in diabetic patients and 75% in healthy subjects (Figure 4 and Figure 5). Out of these, the proportion that was deemed to be granules due to their shape and absence of a motion contrast signal was 18% and 19%, in the respective groups. The majority of elements, however, were elongated with an evaluable motion contrast signal throughout their extensions in the adjacent B-scans and hence were classified as perfused filiform elements, namely 67% in diabetic patients and 81% in healthy subjects. Non-perfused filiform elements, defined by the absence of a motion contrast signal or the signal being consequently located beside them, were only seen in diabetic patients, in whom they constituted on average 15% of the classifiable dots. Up to 56% of the elements with a motion contrast signal were susceptible to projection artifacts from the superficial capillary plexus.

Hyperreflective dots in the outer nuclear layer were examined by the inspection of all 131 structural B-scans of the macula in all 16 participants. Healthy subjects had from 0 to 3 (median 0.5) hyperreflective dots in the outer nuclear layer and diabetic patients had from 0 to 11 dots (median 2.0) (Table 2). The hyperreflective dots measured around 15–20 µm in the axial direction, on average from 11 to 39 µm in the transverse direction, and none of them displayed a motion contrast signal on the angiographic B-scans. Thus, these dots were all unambiguously classified as granules.

## 4. Discussion

This study examined hyperreflective dots in the inner and outer nuclear layer of the retina in patients with mild NPDR or quiescent PDR and, for comparison, in healthy subjects. By an analysis and correlation of densely spaced structural and angiographic OCT B-scans, the dots were characterized by number, location, diameter and, when they could be seen to be part of hyperreflective elements that spanned multiple adjacent OCT B-scans, by length, which enabled a description of shape, and, finally, by their perfusion status.

In both diabetic patients and healthy subjects, the majority of hyperreflective dots in the inner nuclear layer formed parts of elongated elements, the characteristics of which were clearly compatible with those of retinal capillaries: They had a diameter of 10–25 µm, could be followed for a distance of 51 to 183 µm, and the majority were shown to have a motion contrast signal and were thus classified as perfused filiform structures (46% in diabetic patients and 61% in healthy subjects). Also, the location of these elements supported that they mainly represented capillaries from the deep plexus seen in cross-section [15]. Remarkably, 10% of the hyperreflective elements in the diabetic patients, in both mild NPDR and quiescent post-photocoagulation PDR, were non-perfused or, at least, perfused at a rate too low to be detectable, and were classified as non-perfused filiform elements, whereas no such structures were found in the healthy subjects (0%). These non-perfused filiform elements could be occluded or non-perfused acellular capillaries, possibly with thickened basement membranes, as has been observed in histological samples of diabetic retinopathy [14,16]. Capillary thickening may explain the prominence of the dots in diabetic patients compared to healthy subjects. Alternatively, we speculate that the elements could represent beta-amyloid [17]. All participants also had granular structures without signs of perfusion in the inner nuclear layer (12.5% in diabetic patients and 14% in healthy subjects), and a considerable proportion of unclassifiable dots that could not be traced on the angiographic OCT scans (31.5% and 25%, respectively).

In the outer nuclear layer, the very few hyperreflective dots were all classified as granules, given that they measured 15–20 µm in the axial direction and 11–39 µm in the transverse, which were compatible with a rounded shape, and had no sign of perfusion. This suggests that the granules in both the inner and outer nuclear layer may represent cells, be they single cells or clusters of cells.

Previous theories about the nature and origin of hyperreflective dots in the retina have mainly concerned diabetic macular edema and age-related macular degeneration, in which they have been suggested to represent precursors to hard exudates [1,5]. Hyperreflective dots in the outer nuclear layer in macular edema have been shown to be associated with a poor visual prognosis [18], and the numbers correlate with total macular volume [19]. The present study has the virtue, in this context, that it excluded eyes with macular edema or hard exudate. Compared to hard exudates on OCT, the dots of the present study were less hyperreflective and did not cast a shadow on the posterior layers of the retina and choroid. Systemic factors associated with the number of hyperreflective dots include higher total and/or low-density lipoprotein cholesterol [19] and poor-quality glycometabolic control, but not duration of diabetes or age [20].

It has been proposed that hyperreflective dots represent inflammatory cells, perhaps in the form of activated microglia in diabetic retinopathy [6,20]. Similar inner nuclear layer granules in patients with retinitis pigmentosa and healthy subjects have been suggested to represent degenerated Müller cells [7]. As in the present study, their most common location was at the border to the outer plexiform layer [7]. These studies reported fewer dots than in the present study, which could be explained by use of an older OCT device than in the present study or the use of less scan-averaging. A study analyzing en face angiograms of OCT angiography proposed that hyperreflective foci represent lipid-laden macrophages attached to vessel walls [21]. Furthermore, one study determined a hyperreflective dot in the vitreous of a rat to be a macrophage using a light microscope [22].

Several recent studies have proposed semi-automatic or automatic approaches for the characterization of hyperreflective dots. Again, focus has mostly been on macular edema [23], but also on patients with retinopathy without edema [24,25]. Despite the emergence of angiographic OCT and improved automatic identification, it has, to the best of our knowledge, still not been demonstrated that many of the hyperreflective dots in the inner nuclear layer are simply capillaries, as we do in this study. We suggest that the reason why capillaries in an OCT B-scan do not cast a backshadow, as examined in previous studies [4], is that only one red blood cell passes at a time, giving too little reflectivity to cause it.

Our study has obvious limitations. First of all, the small size of the hyperreflective dots approached the edge of what the OCT instrument could resolve and quantify, and their prominence seemed to vary with the angle at which the OCT light beam fell on them during scan acquisition. Second, the manual analysis procedure of the structural B-scans was susceptible to subjective influences. The variation in diameter and intensity of the dots could not be accurately assessed with the proprietary software, and standardized segmentation of the irregular border of the inner nuclear layer was difficult. Third, it was uncertain whether the two types of B-scans were obtained from exactly the same location of the retina. Since our scanning protocol is time-consuming, the scans were susceptible to motion artifacts due to head movement during or between the two scan sessions, causing the images to rotate differently along the axial axis, or due to small saccades or the slow drift of the eye [26]. The two types of scans could not overlap fully or be made as follow-up. Fourth, the assessment of the angiographic B-scans was limited by the low averaging of seven B-scans, making it difficult to distinguish dots from background speckle noise. Lack of perfusion could be due to a blood flow lower or higher than what the OCT device could detect. It could not be determined if a flow signal seen besides a dot was derived from the dot itself or solid structures attached to its edges, as described for lipoproteins [26]. These reasons could partly explain the high proportion of unclassifiable dots. The degree of projection artifacts from the superficial capillary plexus is uncertain. No quantitative measures could be provided automatically.

Due to the small study size and the lack of blinding, observer bias may have influenced this classification of hyperreflective dots. Nevertheless, Vujosevic et al. found excellent intra- and intergrader repeatability of a comparable method of manual identification of similar dots [4].

Performing a valid analysis of microstructures against a granular background may benefit from further improvement of OCT hardware and analytical tools. We believe that a particularly promising clinical potential lies in the development of an automated tool for the mapping of capillary occlusion and other types of capillary derangement in vivo.

## Figures and Tables

**Table 1:** Characteristics of study population.

Subject (Group)	Age	Sex	Eye	Diabetic Retinopathy Stage	Diabetes Mellitus Type 1 Duration in Years	Diabetic Retinopathy Duration in Years	Treatment for Diabetic Retinopathy	Diabetic Nephropathy Duration in Years	BCVA (Snellen)
1 (DR)	47	M	L	mNPDR	20	2	None	2	20/16
2 (DR)	56	M	R	mNPDR	44	2	None	0	20/20
3 (DR)	29	M	L	mNPDR	17	2.5	None	0	20/20
4 (DR)	66	F	L	mNPDR	22	1	None	0	20/25
5 (DR)	29	M	R	mNPDR	17	2	None	0	20/16
6 (DR)	48	F	L	QPDR	31	18	PRP	18	20/20
7 (DR)	47	M	R	QPDR	37	12	PRP	0	20/16
8 (DR)	51	M	R	QPDR	38	22	PRP	6	20/16
9 (C)	47	F	L	-	-	-	None	-	-
10 (C)	60	F	R	-	-	-	None	-	-
11 (C)	28	M	L	-	-	-	None	-	-
12 (C)	63	F	L	-	-	-	None	-	-
13 (C)	23	M	R	-	-	-	None	-	-
14 (C)	48	M	R	-	-	-	None	-	-
15 (C)	46	F	L	-	-	-	None	-	-
16 (C)	51	F	R	-	-	-	None	-	-

Note: Subject 1 and 6 had received a simultaneous pancreas and kidney transplantation 0.25 and 1 year prior to the eye examination, respectively. Abbreviations: DR: type 1 diabetes patient with diabetic retinopathy. C: healthy control subject. M: male. F: female. L: left. R: right. mNPDR: mild non-proliferative diabetic retinopathy. QPDR: quiescent proliferative diabetic retinopathy. PRP: panretinal photocoagulation. BCVA: best-corrected visual acuity.

**Table 2:** Hyperreflective dot characteristics on structural OCT B-scans; analysis of the parafoveal inner nuclear layer on a single B-scan; analysis of the full outer nuclear layer on 131 contiguous B-scans at 11 µm distance covering a 15-by-5-degree field.

	Diabetic Patients									Healthy Controls									
	Mild NPDR					Quiescent PDR													
	1	2	3	4	5	6	7	8	Median	9	10	11	12	13	14	15	16	Median	*p*-Value ***
	Inner nuclear layer *																		
Hyperreflective dots (no.)	18	14	13	13	7	13	12	15	13	13	8	16	11	13	6	8	7	9.5	0.3
Granules ≤ 3 adjacent B-scans (no.)	5	2	3	4	0	6	2	3	3	2	0	7	0	0	2	3	6	2	0.7
Elongated elements > 3 adjacent B-scans (no.)	13	12	10	9	7	7	10	12	10	11	8	9	11	13	4	5	1	8.5	0.57
Transversal dimension of elongated (mean in µm)	59	79	51	67	115	63	183	82	73	128	87	76	180	121	58	62	66	81.5	0.48
Location near inner plexiform layer (no. (%))	7 (39)	6 (43)	6 (46)	4 (31)	2 (29)	2 (15)	1 (8)	6 (40)	5 (31)	4 (31)	0 (0)	3 (19)	2 (18)	4 (31)	0 (0)	3 (38)	3 (43)	3 (23)	0.106
Location inside inner nuclear layer (no. (%))	3 (17)	0 (0)	1 (8)	4 (31)	0 (0)	7 (54)	3 (25)	7 (47)	3 (23)	0 (0)	1 (13)	1 (6)	0 (0)	0 (0)	1 (17)	0 (0)	0 (0)	0 (5)	0.013
Location near outer plexiform layer (no. (%))	8 (44)	8 (57)	6 (46)	5 (38)	5 (71)	4 (31)	8 (67)	2 (13)	5.5 (46)	9 (69)	7 (88)	12 (75)	9 (82)	9 (69)	5 (83)	5 (63)	4 (57)	8 (73)	0.29
	Outer nuclear layer **																		
Hyper-reflective dots (no.)	2	0	11	2	2	4	1	3	2	0	0	0	3	1	0	1	1	0.5	
Transversal dimension (mean in µm)	11	-	24	39	11	30	22	29	23	-	-	-	11	22	-	22	33	5.5	

Abbreviations: NPDR = non-proliferative diabetic retinopathy. PDR = proliferative diabetic retinopathy. * Results for the inner nuclear layer from the analysis of a 1000 µm parafoveal area in one transfoveal B-scan. ** Results for the outer nuclear layer from the analysis of all 131 B-scans. *** Diabetic patients vs. healthy controls.

## Data Availability

Data underlying the results presented in this paper are available in the tables and figures.
